# Comparison of the clinical efficacy and pharmacoeconomics of tofacitinib and adalimumab in Chinese patients with rheumatoid arthritis: An analysis based on propensity score matching

**DOI:** 10.1007/s10067-025-07431-x

**Published:** 2025-04-29

**Authors:** Qin-yao Xu, Ya-qian Liu, Wan-qiu Tong, Yan-ran Yin-ruo, Sheng-qian Xu, Zong-wen Shuai

**Affiliations:** 1 Department of Rheumatology & Immunology, The First Affiliated Hospital of Anhui Medical University, No. 218, Ji-Xi Road, Hefei, 230022 China; 2 Department of Rheumatology & Immunology, Anqing First People’s Hospital of Anhui Medical University, Anqing, 246003 China

**Keywords:** Adalimumab, Cost-effectiveness, Pharmacoeconomics, Rheumatoid arthritis, Tofacitinib

## Abstract

**Objective:**

This study aimed to investigate the clinical efficacy and pharmacoeconomics of tofacitinib versus adalimumab in treating patients with rheumatoid arthritis (RA).

**Methods:**

Propensity score matching was used to obtain matched cohorts of 116 RA patients treated with tofacitinib or adalimumab. Clinical and laboratory indicators were compared before and after treatment between the two groups. A Markov model was used to assess cost-effectiveness, incorporating direct and indirect costs. Sensitivity analyses validated model stability, with a 6-month cycle simulating lifelong disease progression (27 years).

**Results:**

Both the tofacitinib-treated group and the adalimumab-treated group showed significant improvements in terms of swollen and tender joint counts, duration of morning stiffness, VAS and HAQ scores, and ESR, CRP, and DAS28 levels (*P* < 0.05). Adalimumab treatment resulted in reductions in rheumatoid factor levels (*P* < 0.05). ACR20 (χ^2^ = 0.240, *P* = 0.624), ACR50 (χ^2^ = 0.321, *P* = 0.571), and ACR70 (χ^2^ = 0.222, *P* = 0.637) response rates did not differ significantly between groups. Adverse events included tuberculosis, leukopenia, mild liver dysfunction in the adalimumab group, and herpes zoster and mild liver dysfunction in the tofacitinib group, with gastrointestinal reactions observed in both groups (*P* > 0.05). Cost-effectiveness analysis indicated that tofacitinib was more cost-effective than adalimumab. Univariate sensitivity analysis identified ACR50 and ACR70 response rates as key influencing factors, while probabilistic sensitivity analysis showed a 99.38% probability of tofacitinib being cost-effective relative to adalimumab.

**Conclusion:**

Tofacitinib combined with methotrexate demonstrated comparable clinical efficacy and safety to adalimumab combined with methotrexate, with better cost-effectiveness, supporting its use as a favorable treatment strategy.

**Table:** 

**Key Points** • *Our research indicates that tofacitinib exhibits a more favorable pharmacoeconomic profile for treating rheumatoid arthritis, characterized by reduced treatment costs and increased quality-adjusted life years*. • *Tofacitinib consistently demonstrated its cost-effectiveness superiority over adalimumab across various sensitivity analyses, exhibiting a 99.38% likelihood of being more cost-effective*.

## Introduction

Rheumatoid arthritis (RA) is a chronic autoimmune inflammatory disease with an increasing global prevalence [1]. It is characterized by synovial inflammation and progressive cartilage and bone destruction, primarily affecting the joints. Patients commonly experience joint pain, morning stiffness, and gradual joint deterioration, which may lead to disability [2–4]. Long-term pharmacological therapy is essential for RA management. Since 1996, the American College of Rheumatology (ACR) has emphasized the importance of early diagnosis and treatment to slow disease progression and delay joint damage [5, 6]. Delayed or inadequate treatment increases the risk of joint damage, impairing ability to work and quality of life while imposing a significant economic burden on patients, their families, and society [7–10].

Pharmacological treatments for RA include conventional disease-modifying antirheumatic drugs (cDMARDs), such as methotrexate (MTX) and leflunomide; biologic disease-modifying antirheumatic drugs (bDMARDs), such as tumor necrosis factor (TNF)-α inhibitors; and targeted synthetic disease-modifying antirheumatic drugs (tsDMARDs), such as Janus kinase (JAK) inhibitors [11]. Both adalimumab (ADA) and tofacitinib (TOF) belong to these categories and have demonstrated efficacy and safety [12–14]. The Oral RA (ORAL) trial, conducted across multiple centers, compared the efficacy of these medications and reported similar results [15, 16]. However, limited research has examined the comparative efficacy and safety of ADA and TOF in RA treatment within China. RA treatment often involves a combination of these drugs. Although bDMARDs are associated with high treatment costs, they provide greater efficacy when the clinical response to cDMARDs is poor. The ACR and the European League Against Rheumatism (EULAR) guidelines recommend treatment escalation to bDMARDs or tsDMARDs for patients with RA who show poor response to cDMARDs such as MTX [17, 18]. With economic growth, an increasing number of RA patients worldwide, including in China, are opting for bDMARDs as a treatment modality [19].

As RA is a chronic inflammatory disease requiring long-term treatment, evaluating the cost-effectiveness of biologic agents is crucial [20]. Smolen et al. [21] noted in the 2019 EULAR recommendations for the management of RA that the condition was associated with high personal, medical, and social costs, and physicians should consider these factors when selecting treatments. With an increase in global economic levels, healthcare expenditures are also increasing. Thus, the efficient allocation of medical resources remains a significant challenge in China and worldwide. Pharmacoeconomics provides a systematic approach for comparing the economic costs and overall benefits of medical treatments, aiding in evidence-based decision-making to optimize treatment strategies [22]. Several pharmacoeconomic studies from the USA and Taiwan [23, 24] have indicated that TOF is a cost-effective option. In 2021, Tan et al. [25] at the Second Xiangya Hospital in Hunan, China, conducted a cost-effectiveness analysis of TOF combined with MTX for RA treatment from the perspective of the Chinese healthcare system, employing a patient-level simulation model. Their findings demonstrated that TOF provided greater benefits at a lower cost in both first- and second-line settings, establishing its dominance in clinical treatment. In third- and fourth-line settings, TOF offered additional benefits with incremental costs of $333.73 and $9,669.34 per quality-adjusted life year (QALY), respectively. TOF was deemed cost-effective at a willingness-to-pay threshold of $10,378 per QALY, aligning with China's 2019 per capita GDP. However, limited data on its cost-effectiveness in real-world clinical practice in mainland China is available.

This study compares the clinical and economic effects of two DMARD-based RA treatment strategies: TOF combined with MTX and ADA combined with MTX. Understanding the pharmacoeconomic impact of these treatment modalities is essential for healthcare providers and policymakers to optimize patient outcomes while managing costs.

## Materials and methods

### Patients

This study was approved by the Ethics Committee (Approval Number: 20121090). Written informed consent was obtained from all patients before enrollment. A total of 200 patients with RA who did not respond to cDMARDs, including oral MTX (10 mg/week) for at least six months, were recruited from the Rheumatology and Immunology Department of the First Affiliated Hospital of Anhui Medical University between March 2020 and October 2022. Among them, 100 patients received TOF combined with MTX, while the remaining 100 received ADA combined with MTX. The patients'ages ranged from 18 to 86 years, with a mean age of 52 years. The body mass index (BMI) ranged from 13.33 to 32.14 kg/m^2^, with a mean BMI of 22.30 kg/m^2^. The proportions of female participants in the TOF and ADA groups were 86.00% and 88.00%, respectively. All patients met the revised 1987 ACR diagnostic criteria for RA as well as the 2010 ACR/EULAR classification criteria.

Exclusion criteria included severe cardiovascular diseases, hepatic dysfunction, renal impairment, hematologic disorders, malignancies, hepatitis B, tuberculosis, or other chronic diseases. Study termination criteria included voluntary withdrawal of patients, severe adverse reactions, or worsening of other underlying conditions due to treatment.

### Efficacy and safety

The treatment regimen involved TOF and ADA administration for six months. Clinical and laboratory assessments included general characteristics (age, sex, BMI, and disease duration) and disease activity indicators, including swollen joint count (SJC), tender joint count (TJC), duration of morning stiffness, visual analog scale (VAS) score, Health Assessment Questionnaire (HAQ) score, and Disease Activity Score in 28 Joints (DAS28). Laboratory parameters included erythrocyte sedimentation rate (ESR), C-reactive protein (CRP), and rheumatoid factor (RF) levels. Clinical response was evaluated based on ACR20, ACR50, and ACR70 response rates.

The incidence and severity of adverse events were systematically recorded to assess the safety profile of TOF compared with ADA over the six-month study period. Clinical laboratory tests, vital sign assessments, and physical examinations were conducted at scheduled visits to ensure comprehensive monitoring of participant safety throughout the study period.

### Pharmacoeconomic model structure

A decision-analytic Markov model was used to perform the pharmacoeconomic analysis, simulating the long-term effects of TOF compared with ADA for RA treatment from a societal perspective. Clinical efficacy data included changes in HAQ scores based on ACR response, transition probabilities, and the proportion of nonresponders. Utility values were obtained from relevant studies, incorporating indicators from the study by Becky et al. [25]. The pharmacoeconomic analysis used a decision-analytic Markov model that integrated comprehensive pharmacoeconomic data from each patient, including total costs, direct medical costs, medication costs, state costs, direct nonmedical costs, indirect costs, and QALYs. The analysis also assessed the QALY difference (TOF vs. ADA), cost difference (TOF vs. ADA), and the incremental cost-effectiveness ratio (ICER). Sensitivity analyses were conducted to evaluate the stability of the model, with univariate sensitivity analysis performed for key variables, including cost, efficacy, and utility values.

Based on the clinical treatment route used in China for RA, with reference to the study by Fatemi et al. [26], the model was structured with a cycle length of 6 months and simulated the lifelong progression of the disease over 27 years. Given that treatment typically begins at age 51 and the average life expectancy in China is 78 years, the model allowed simulation of the lifelong progression of patients over 27 years.

### Statistical analysis

Data analysis was conducted using SPSS 27.0. Normally distributed continuous variables are presented as the mean ± standard deviation (SD), while nonnormally distributed variables are reported as the median (interquartile range). Categorical variables were compared using χ^2^ tests, and continuous variables were analyzed using *t*-tests or nonparametric tests as appropriate. A *P* value < 0.05 was considered statistically significant.

## Results

### Baseline clinical characteristics

Before propensity score matching, the TOF group had a disease duration of 7.65 (2.43–14.75) years, a DAS28 score of 3.25 ± 1.24, an SJC of 1.00 (0.00–2.00), a TJC of 1.00 (0.00–5.75), a morning stiffness duration of 0.00 (0.00–20.00) min, a VAS score of 4.00 (3.00–5.00), a HAQ score of 0.20 (0.00–0.75), an ESR of 23.00 (14.25–44.50) mm/h, and a CRP level of 5.18 (2.13–12.78) mg/L. All these values were significantly lower than those in the ADA group, which had a disease duration of 9.50 (3.00–18.97) years, a DAS28 score of 4.36 ± 1.17, an SJC of 2.50 (1.00–6.00), a TJC of 5.00 (2.00–10.00), a morning stiffness duration of 10.00 (0.00–60.00) min, a VAS score of 6.00 (5.00–7.00), a HAQ score of 0.88 (0.45–1.50), an ESR of 34.00 (22.00–63.00) mm/h, and a CRP level of 30.69 (12.80–73.42) mg/L (*P* < 0.05). No significant differences between the two groups were observed in age, female proportion, or BMI. These results indicate that disease activity was significantly lower in the TOF treatment group than in the ADA treatment group Table 1.

**Table 1:** Baseline characteristics of patients before propensity score matching

	TOF (*n* = 100)	ADA (*n* = 100)	*P*
Age (year)	51.5 (43.50 ~ 57.10)	52.00 (43.25 ~ 58.75)	0.990
Female (%)	86.00	88.00	0.817
BMI (kg/m^2^)	22.00 ± 3.31	21.83 ± 2.65	0.644
Disease Duration (year)	7.65 (2.43 ~ 14.75)	9.50 (3.00 ~ 18.97)	0.001
SJC	1.00 (0.00 ~ 2.00)	2.50 (1.00 ~ 6.00)	0.001
TJC	1.00 (0.00 ~ 5.75)	5.00 (2.00 ~ 10.00)	0.001
Morning stiffness (min)	0.00 (0.00 ~ 20.00)	10.00 (0.00 ~ 60.00)	0.001
VAS	4.00 (3.00 ~ 5.00)	6.00 (5.00 ~ 7.00)	0.001
ESR (mm/h)	23.00 (14.25 ~ 44.50)	34.00 (22.00 ~ 63.00)	0.001
CRP (mg/L)	5.18 (2.13 ~ 12.78)	30.69 (12.80 ~ 73.42)	0.001
DSA28	3.25 ± 1.24	4.36 ± 1.17	0.001
RF (IU/mL)	56.85 (27.60 ~ 119.40)	198.23 (75.92 ~ 442.27)	0.281
HAQ	0.20 (0.00 ~ 0.75)	0.88 (0.45 ~ 1.50)	0.001

*BMI* Body mass index, *SJC* Swollen joint count, *TJC* Tender joint count, *VAS* Visual analog scale score, *ESR* Erythrocyte sedimentation rate, *CRP* C-reactive protein, *DSA28* Disease activity score in 28 joints score, *RF* Rheumatoid factor, *HAQ* Health assessment questionnaire score

After propensity score matching, 58 patients were included in each group for further analysis. No significant differences were found between the groups in age, sex, disease duration, BMI, SJC, TJC, morning stiffness duration, VAS, ESR, CRP, DAS28, RF, or HAQ score, indicating good comparability between the two groups (Table 2).

**Table 2:** Baseline characteristics of patients after propensity score matching

	TOF (*n* = 58)	ADA (*n* = 5)	*P*
Age (year)	50.28 ± 13.10	51.36 ± 12.77	0.652
Female (%)	89.66 (52/58)	86.21 (50/58)	0.57
Disease Duration (year)	5.50 (2.00 ~ 12.00)	5.00 (0.79 ~ 11.25)	0.381
BMI (kg/m^2^)	21.97 ± 3.36	22.01 ± 2.70	0.851
SJC	2.00 (0.00 ~ 5.00)	2.00 (0.75 ~ 4.00)	0.849
TJC	4.50 (1.00 ~ 10.00)	3.00 (1.00 ~ 7.25)	0.681
Morning stiffness (min)	5.00 (0.00 ~ 52.50)	5.00 (0.00 ~ 60.00)	0.835
VAS	4.50 (3.00 ~ 6.00)	5.00 (4.00 ~ 6.00)	0.357
ESR (mm/h)	28.50 (16.75 ~ 51.75)	31.00 (20.00 ~ 47.00)	0.671
CRP (mg/L)	7.45 (2.98 ~ 16.59)	6.80 (3.13 ~ 21.45)	0.96
DSA28	3.90 ± 1.18	3.94 ± 1.04	0.841
RF (IU/mL)	70.80 (26.30 ~ 124.58)	63.45 (25.00 ~ 106.13)	0.556
HAQ	0.52 (0.00 ~ 0.96)	0.63 (0.23 ~ 1.10)	0.238

*BMI* Body mass index, *SJC* Swollen joint count, *TJC* Tender joint count, *VAS* Visual analog scale score, *ESR* Erythrocyte sedimentation rate, *CRP* C-reactive protein, *DSA28* Disease activity score in 28 joints score, *RF* Rheumatoid factor, *HAQ* Health assessment questionnaire score

### Comparison of efficacy between TOF and ADA treatments

**Fig. 1:**
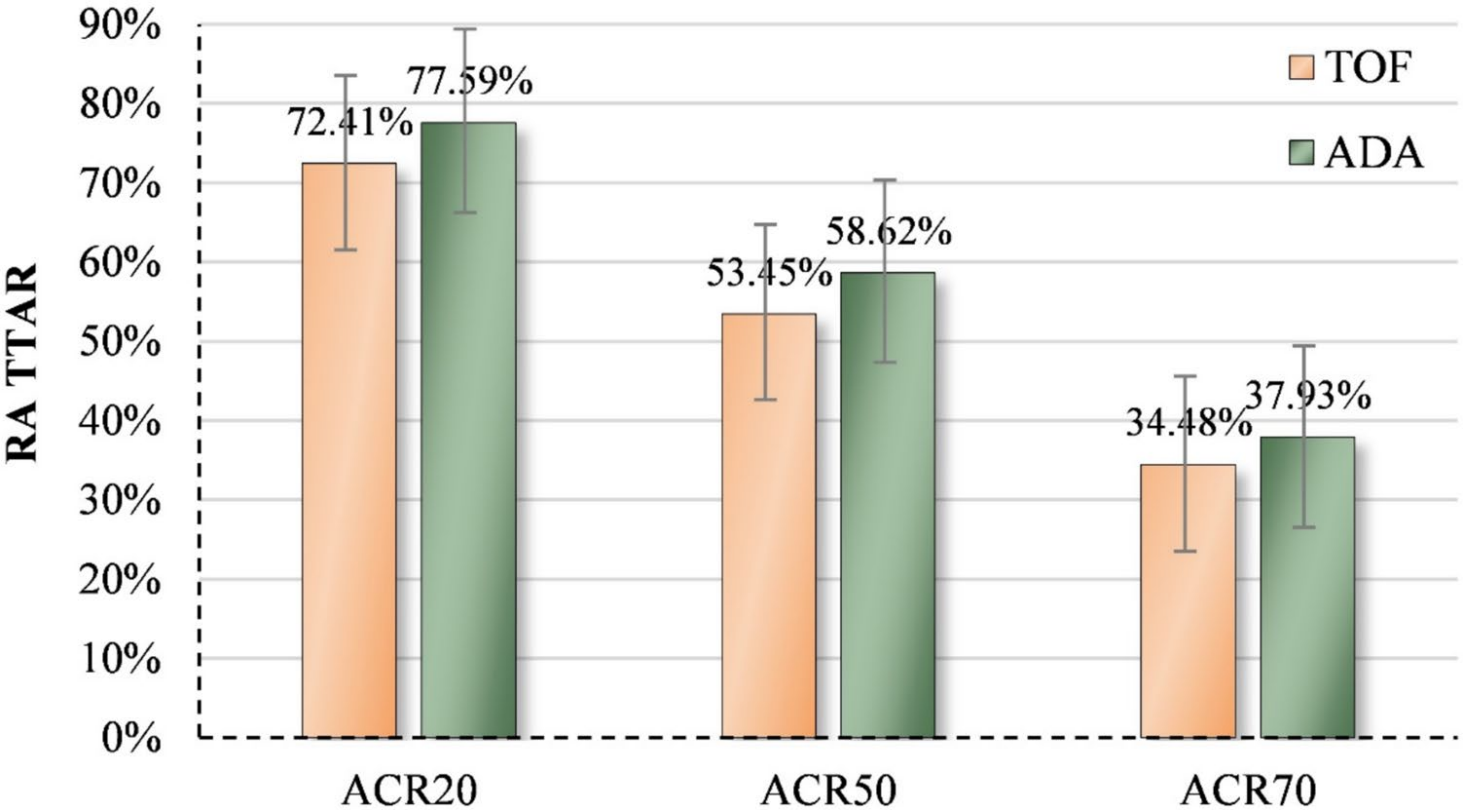
Six-month treat-to-target outcomes in the TOF and ADA groups for the treatment of RA

After 6 months of treatment, both the TOF and ADA groups showed significant improvements in clinical indicators, including SJC, TJC, morning stiffness duration, VAS score, HAQ score, ESR, CRP, and DAS28 score. However, RF levels did not significantly improve in the TOF group (Table 3), whereas significant improvements were observed in the ADA group (Table 4). No significant differences were observed between the TOF and ADA groups in ΔSJC, ΔTJC, Δmorning stiffness duration, ΔVAS score, ΔHAQ score, ΔESR, ΔCRP, or ΔDAS28 score. However, ΔRF improvement was significantly higher in the ADA group than in the TOF group (Table 5). The TOF group had ACR20, ACR50, and ACR70 response rates of 72.41% (42/58), 53.45% (31/58), and 34.48% (20/58), respectively, while the ADA group had response rates of 77.59% (45/58), 58.62% (34/58), and 37.93% (22/58), respectively. There were no significant differences between the TOF and ADA groups in ACR20 (χ^2^ = 0.240, *P* = 0.624), ACR50 (χ^2^ = 0.321, *P* = 0.571), or ACR70 (χ^2^ = 0.222, *P* = 0.637) response rates (Fig. 1).

**Table 3:** Comparison of the clinical efficacy of the TOF and ADA groups in the treatment of RA

	TOF(*n* = 58)	ADA (*n* = 58)	z/t	*P*
ΔSJC	1.00 (0.00 ~ 4.00)	2.00 (0.00 ~ 3.00)	0.545	0.586
ΔTJC	2.00 (0.00 ~ 6.00)	2.00 (1.00 ~ 5.25)	0.649	0.516
ΔMorning stiffness (min)	0.00 (0.00 ~ 17.50)	0.50 (0.00 ~ 30.00)	0.817	0.414
ΔVAS	1.50 (0.00 ~ 3.00)	2.50 (1.00 ~ 4.00)	3.413	0.231
ΔESR (mm/h)	9.00 (1.00 ~ 20.00)	13.00 (3.75 ~ 20.00)	0.583	0.560
ΔCRP (mg/L)	3.44 (0.83 ~ 11.45)	5.31 (0.66 ~ 12.76)	0.629	0.529
ΔDSA28	1.23 ± 1.41	1.34 ± 1.12	4.103	0.666
ΔRF (IU/mL)	2.75 (− 8.73 ~ 30.10)	21.35 (5.78 ~ 40.80)	2.615	0.009
ΔHAQ	0.52 (0.00 ~ 0.93)	0.58 (0.14 ~ 1.2)	4.693	0.342

*BMI* Body mass index, *SJC* Swollen joint count, *TJC* Tender joint count, *VAS* Visual analog scale score, *ESR* Erythrocyte sedimentation rate, *CRP* C-reactive protein, *DSA28* Disease activity score in 28 joints score, *RF* Rheumatoid factor, *HAQ* Health assessment questionnaire score

**Table 4:** Comparison of clinical efficacy in RA patients treated with ADA before and after 6 months

	ADA Group Treatment at Month 0	ADA Group Treatment at Month 6	z/t	*P*
SJC	2.00 (0.75 ~ 4.00)	0.00 (0.00 ~ 1.00)	4.535	0.001
TJC	3.00 (1.00 ~ 7.25)	0.00 (0.00 ~ 2.00)	5.353	0.001
Morning stiffness (min)	5.00 (0.00 ~ 60.00)	0.00 (0.00 ~ 5.00)	3.481	0.001
VAS	5.00 (4.00 ~ 6.00)	2.50 (1.75 ~ 4.00)	6.211	0.001
ESR (mm/h)	31.00 (20.00 ~ 47.00)	17.00 (10.75 ~ 29.50)	4.526	0.001
CRP (mg/L)	6.80 (3.13 ~ 21.45)	2.05 (0.90 ~ 4.91)	4.571	0.001
DSA28	3.94 ± 1.04	2.60 ± 0.92	9.127	0.001
RF (IU/mL)	63.45 (25.00 ~ 106.13)	32.3 (16.73 ~ 90.15)	4.572	0.001
HAQ	0.63 (0.23 ~ 1.10)	0.05 (0.00 ~ 0.40)	5.424	0.01

*SJC* Swollen joint count, *TJC* Tender joint count, *VAS* Visual analog scale score, *ESR* Erythrocyte sedimentation rate, *CRP* C-reactive protein, *DSA28* Disease activity score in 28 joints score, *RF* Rheumatoid factor, *HAQ* Health assessment questionnaire score

**Table 5:** Comparison of clinical efficacy in RA patients treated with TOF before and after 6 months

	TOF Group Treatment at Month 0	TOF Group Treatment at Month 6	z/t	*P*
SJC	2.00 (0.00 ~ 5.00)	0.00 (0.00 ~ 1.00)	4.137	0.001
TJC	4.50 (1.00 ~ 10.00)	0.00 (0.00 ~ 2.25)	3.935	0.001
Morning stiffness (min)	5.00 (0.00 ~ 52.50)	2.50 (0.00 ~ 11.250	2.58	0.01
VAS	4.50 (3.00 ~ 6.00)	3.00 (1.00 ~ 4.00)	4.624	0.001
ESR (mm/h)	28.50 (16.75 ~ 51.75)	15.00 (10.75 ~ 33.25)	4.284	0.001
CRP (mg/L)	7.45 (2.98 ~ 16.59)	2.20 (0.95 ~ 5.60)	4.309	0.001
DSA28	3.90 ± 1.18	2.66 ± 1.12	6.65	0.001
RF (IU/mL)	70.80 (26.30 ~ 124.58)	54.85 (25.98 ~ 112.83)	1.514	0.13
HAQ	0.52 (0.00 ~ 0.96)	0.00 (0.00 ~ 0.51)	2.207	0.027

*SJC* Swollen joint count, *TJC* Tender joint count, *VAS* Visual analog scale score, *ESR* Erythrocyte sedimentation rate, *CRP* C-reactive protein, *DSA28* Disease activity score in 28 joints score, *RF* Rheumatoid factor, *HAQ* Health assessment questionnaire score

### Comparison of safety between TOF and ADA treatments

During the 6-month treatment period, adverse events were observed in both groups. In the ADA group, one case of tuberculosis, one case of leukopenia, and one case of mild liver function impairment were reported. In the TOF group, one patient developed herpes zoster, and one patient experienced mild liver function impairment Table 6. Furthermore, one case of gastrointestinal reaction occurred in each group (Table 4).

**Table 6:** Incidence of adverse reactions in the TOF and ADA groups (%)

	TOF	ADA	*x*^2^	*P*
Tuberculosis	0	1 (1.7)	1.009	0.315
Herpes zoster	1 (1.7)	0	1.009	0.315
WBC count decreased	0	1 (1.7)	1.009	0.315
Gastrointestinal reactions	1 (1.7)	1 (1.7)	0	1.000
Hepatic insufficiency	1 (1.7)	1 (1.7)	0	1.000

*WBC* White blood cell

### Pharmacoeconomic analysis of tofacitinib versus ADA

#### Base-case analysis of pharmacoeconomics

According to the base-case analysis, the total cost of TOF was ¥144,558, with a lifetime QALY of 10.08, whereas the total cost of ADA was ¥317,747, with a lifetime QALY of 9.86. TOF demonstrated better cost-effectiveness than ADA (Table 7).

**Table 7:** Pharmacoeconomic comparison of TOF and ADA

	TOF	ADA
Total costs	¥144,558	¥317,747
Direct medical costs	¥129,937	¥298,534
Medication costs	¥20,268	¥144,552
State costs	¥109,670	¥153,982
Direct nonmedical costs	¥13,780	¥17,204
Indirect costs	840.93	2008.97
Total QALYs	10.08	9.86
State QALYs	10.08	9.86
Severe Adverse Events QALYs	0.00	0.00
Cost Difference (TOF vs.ADA)	¥− 173,189	
Difference in QALYs (TOF vs.ADA)	0.22	
ICER (TOF vs.ADA)	Absolute advantage	

*QALY* Quality-adjusted life year, *ICER* Incremental cost-effectiveness ratio

The pharmacoeconomic analysis showed that, compared with ADA, TOF had a significant cost advantage, as indicated by significantly lower total costs (¥144,558 vs. ¥317,747) and direct medical costs (¥129,937 vs. ¥298,534). Moreover, treatment with TOF resulted in higher total QALYs (10.08 vs. 9.86) and lower ICERs, supporting its better cost-effectiveness profile.

#### Sensitivity analysis of pharmacoeconomics

**Fig. 2:**
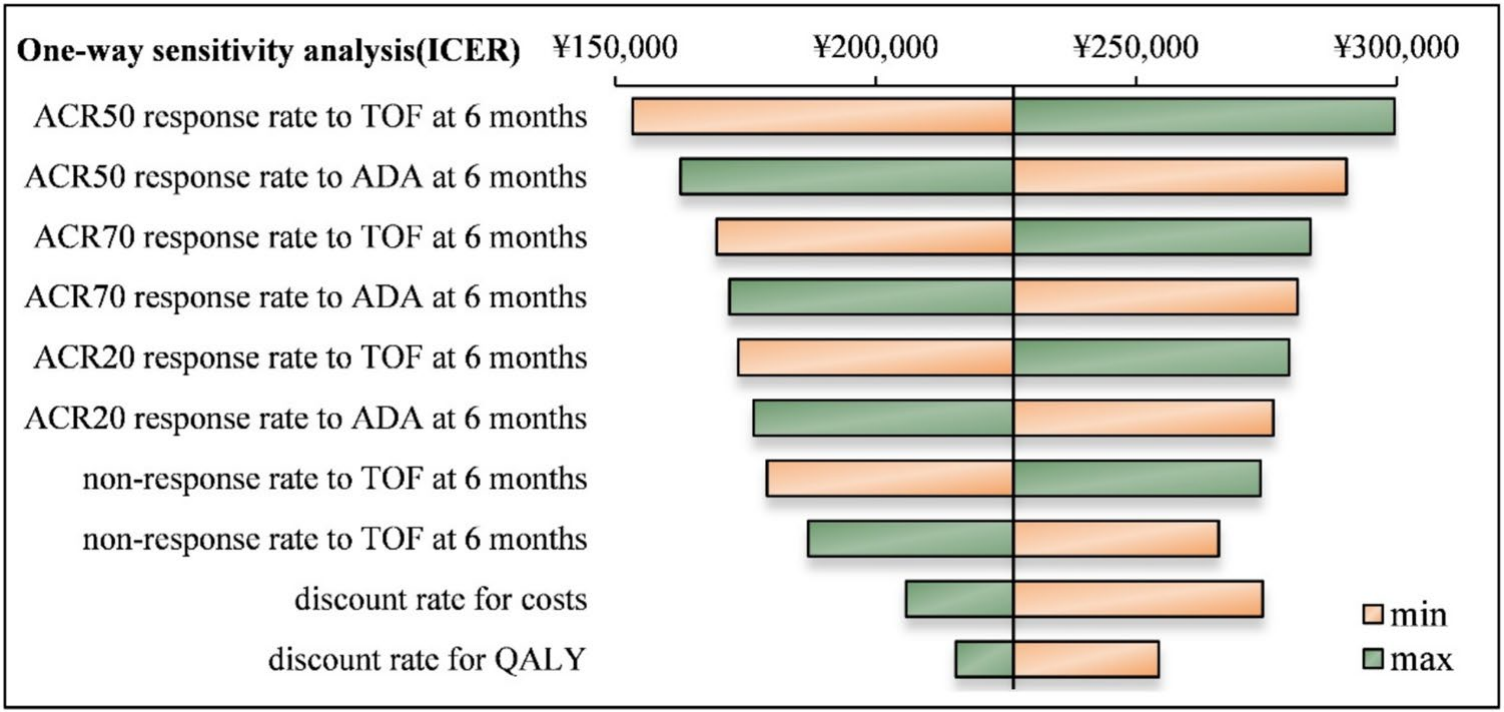
Single-factor sensitivity analysis (cyclone map)

Univariate sensitivity analysis was performed on key model variables, including cost data, efficacy data, and utility values, with each parameter varying by ± 10% from its base value. The three factors with the most significant impact on the analysis were the 6-month ACR50 response rate in the TOF group, the 6-month ACR50 response rate in the ADA group, and the 6-month ACR70 response rate in the TOF group (Fig. 2).

#### Probabilistic sensitivity analysis of pharmacoeconomics

**Fig. 3:**
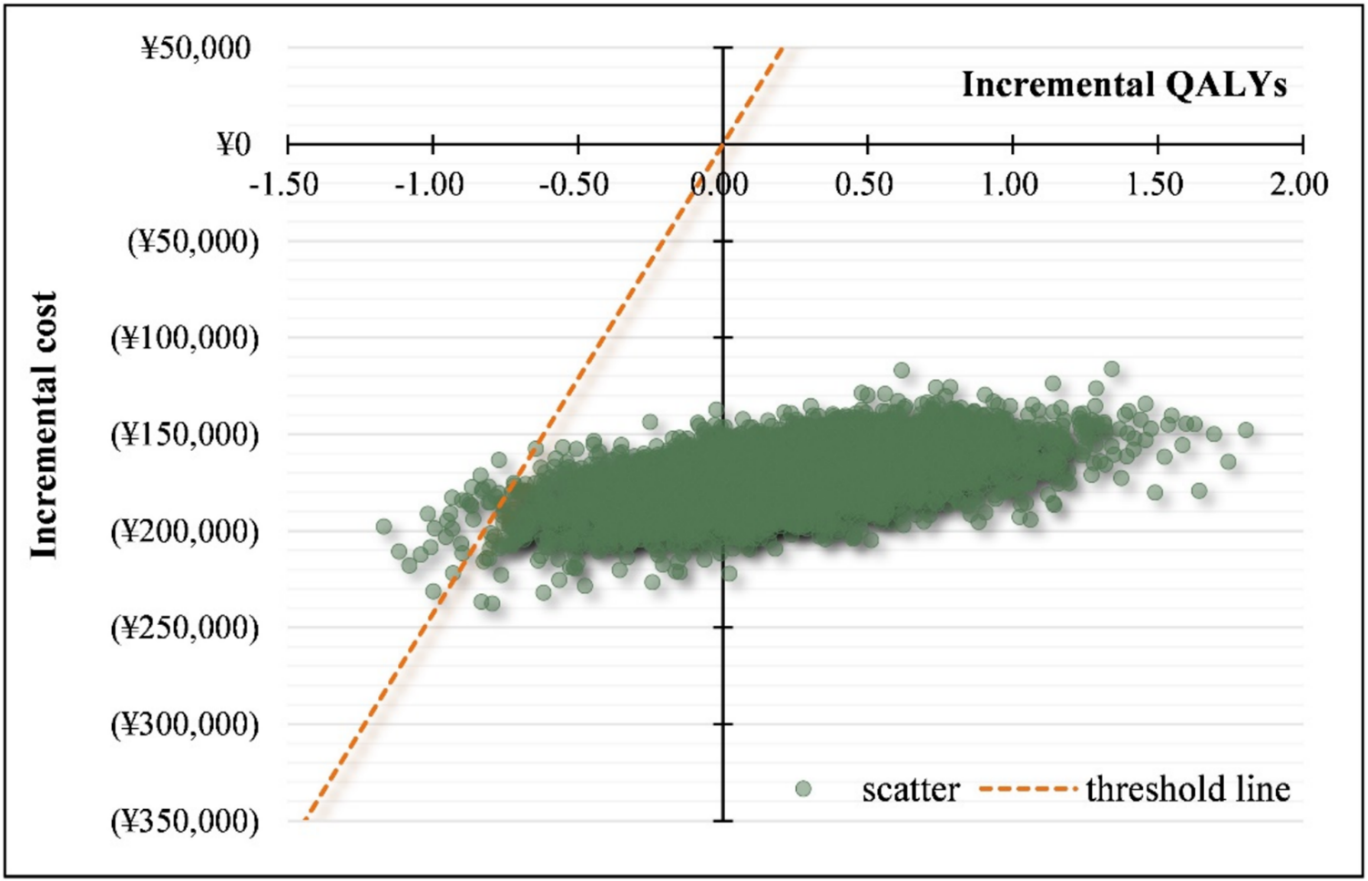
Scatter plot for probabilistic sensitivity analysis of pharmacoeconomics

**Fig. 4:**
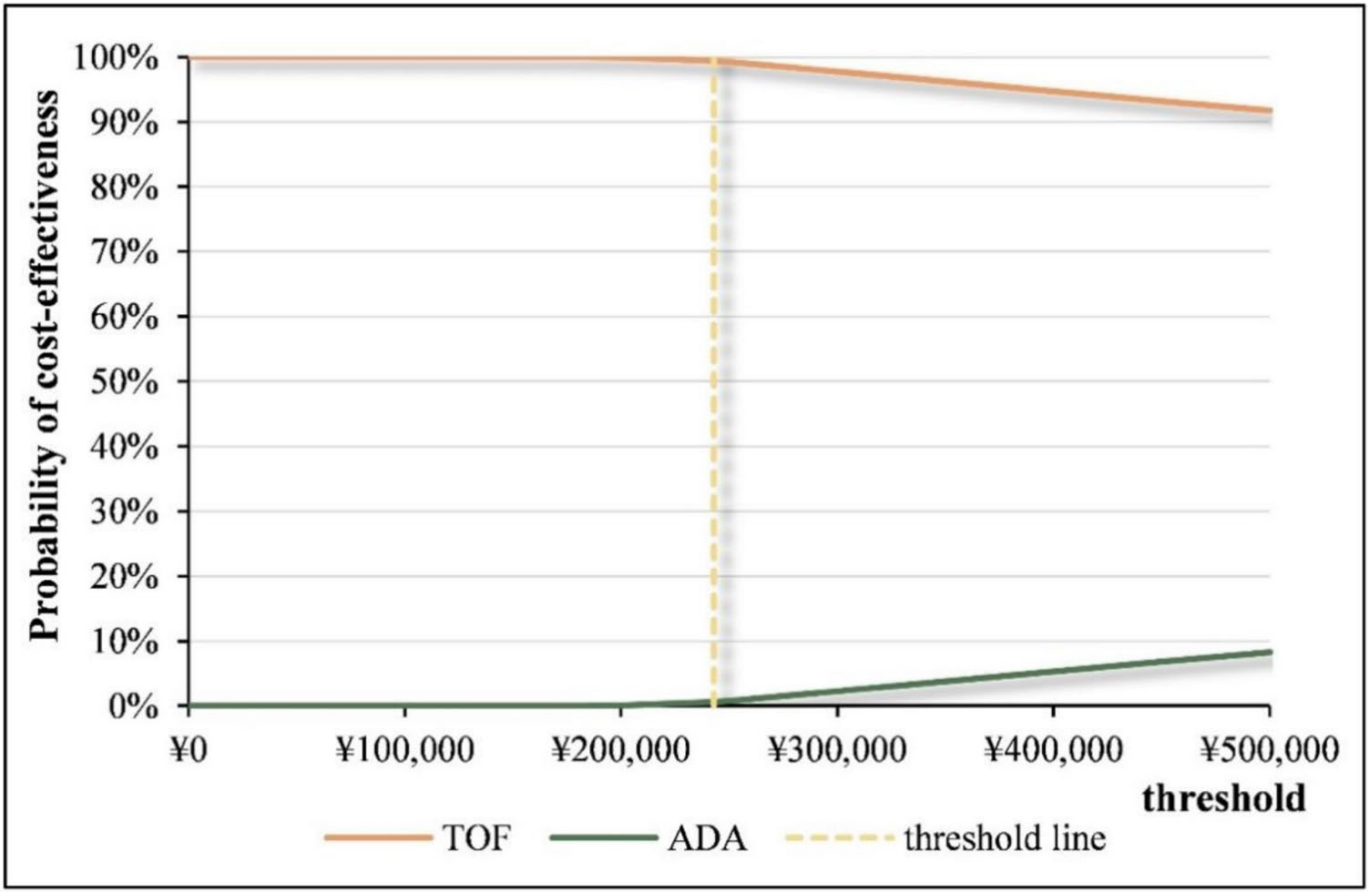
Acceptability curve of cost-effectiveness

For probabilistic sensitivity analysis, costs were assumed to follow a gamma distribution, while utility values and efficacy data followed a beta distribution. The nonparametric bootstrap method was used to conduct 5,000 Monte Carlo simulations, estimating the probability of TOF being more cost-effective than ADA at different willingness-to-pay (WTP) thresholds. Cost-effectiveness acceptability curves were generated based on these simulations. At a WTP threshold of three times the national per capita GDP, the probability of TOF being more cost-effective than ADA was 99.38%, confirming the robustness of the base-case analysis (Figs. 3 and 4).

## Discussion

This study represents one of the first head-to-head real-world evaluations of TOF versus ADA in Chinese patients with RA, addressing a critical evidence gap for Asian populations. Utilizing propensity score matching to address baseline imbalances, similar clinical improvements in SJC, TJC, morning stiffness, VAS, HAQ, ESR, CRP, and DAS28 scores were observed between between the two groups at the six-month mark, consistent with the findings of global phase III clinical trials [27, 28]. Notably, patients in the ADA group showed a greater reductions in RF levels, representing a unique clinical phenotype in this Asian cohort, although this did not result in significant differences in the ACR20/50/70 response rates. These findings confirm the non-inferiority of TOF to ADA previously established in Western populations, while highlighting the unique characteristics of the disease observed in China, such as the lack of RF improvement associated with TOF treatment. Importantly, this real-world analysis provides direct evidence supporting the clinical equivalence of both treatment regimens within the framework of China's healthcare system, where the utilization patterns of methotrexate (MTX) and the accessibility of biologic therapies may differ from those observed in multinational clinical trial settings.

For safety, both treatment groups demonstrated good tolerability. However, one patient in the ADA group developed tuberculosis, one patient experienced leukopenia, and one had mild liver function impairment. In the TOF group, one patient developed herpes zoster infection, and one patient exhibited mild liver function impairment. Moreover, mild gastrointestinal reactions were observed in both groups. Similar findings have been reported in other studies. In a follow-up study of RA patients treated with TOF for up to 8.5 years, Cohen et al. [29] reported that TOF was not only effective but also safe. Similarly, Winthrop et al. [29] observed significant improvements in clinical laboratory indicators, joint swelling and tenderness, and disease activity in RA patients treated with TOF, with a low incidence of adverse events. Burmester et al. [30] conducted a long-term, large-scale clinical trial using data from 71 studies on ADA and found that long-term ADA use in immune-mediated diseases was safe.

The incidence of RA has increased with advancements in medical standards and an increase in public awareness, drawing increasing global attention. Moreover, RA is associated with a significant economic burden [31]. Inadequate symptom control can negatively affect patients'social functioning, work capacity, and overall well-being [32]. Therefore, optimizing RA treatment regimens remains important for healthcare decision-makers worldwide. Pharmacoeconomics plays a key role in guiding treatment selection for RA by optimizing costs, allocating resources efficiently, and maximizing treatment benefits. Patel et al. [33] used a Markov model to estimate the cumulative costs and utilities over the lifespan of patients with RA who had an inadequate response to MTX. Their cost-effectiveness analysis highlighted the economic advantages of early combination therapy with MTX and bDMARDs. Similarly, a study conducted in Spain [34] demonstrated that among patients with moderate-to-severe RA refractory to csDMARDs and those that had previously undergone MTX treatment with inadequate responses to second-line TNF inhibitors, the combination of TOF and MTX resulted in more significant QALY gains, with total cost savings ranging from €5,783 to €13,975. These findings suggest that when introduced as an initial second-line therapy, TOF represents a cost-saving intervention. Cost-effectiveness analyses conducted in the USA [24] further supported the economic advantage of TOF combined with MTX over ADA combined with MTX in RA treatment.

Similarly, a study in Taiwan [23] in 2019 compared the combination of TOF and MTX with ADA and MTX as second-line treatments. The TOF + MTX group had an expected lifespan of 31.95 years, compared with 31.92 years in the ADA + MTX group. The total QALYs per patient were 5.13 in the TOF group and 5.04 in the ADA + MTX group, resulting in incremental benefits of 0.03 life years and 0.09 QALYs. These findings indicate that TOF + MTX is a cost-effective treatment option.

In 2020, the inclusion of TOF in China's national centralized drug procurement system led to a significant price reduction. The integration of real-world treatment patterns and China’s National Healthcare Insurance (NHI) pricing data enabled the demonstration of distinct cost-effectiveness profiles that inform policy decision-making, specifically, that TOF provided more QALYs at a lower total cost and lower ICERs than ADA in RA treatment, supporting its superior cost-effectiveness profile. Sensitivity analysis further supported these findings, with the ACR50 and ACR70 response rates showing significant sensitivity to the outcome. Based on propensity score analysis of real-world clinical data, this research allows for a more accurate assessment of the association between treatment effectiveness and economic benefits.

Although the study findings highlight the advantages of TOF over ADA, several limitations should be acknowledged. First, the utility value data in some studies were primarily derived from foreign populations, which may not fully reflect the Chinese population. Second, due to the lack of relevant cost data for palliative care, some studies assumed that palliative care costs were equivalent to those of nonresponse to ADA treatment, potentially leading to cost estimation bias. Furthermore, this was a single-center study with a relatively small sample size and a relatively short follow-up period, which may have affected the accuracy of the study results. Further multicenter, large-sample studies are needed to more comprehensively evaluate the effectiveness and safety of these treatment regimens.

Despite these limitations, the propensity score-based analysis demonstrated that TOF provided better efficacy and economic benefits than ADA in RA treatment. These findings offer valuable insights for clinicians in developing treatment strategies and contribute to optimizing and individualizing therapeutic approaches for RA management.

## Data Availability

All data generated or analyzed during this study were included in this published article.
